# Emerging trends for open access learning

**DOI:** 10.1186/s41039-015-0010-4

**Published:** 2015-07-15

**Authors:** Rachid Benlamri, Fanny Klett

**Affiliations:** 1 Department of Software Engineering, Lakehead University, Thunder Bay, Ontario P7B-5E1 Canada; 2 German Workforce ADL Partnership Laboratory, 99880 Waltershausen, Germany

**Keywords:** Open learning, open education, competency and skills development

## Abstract

This special issue is dedicated to recent opportunities, trends, and expectations that the emergent number of institutions and governments, exploiting the open learning concept, face in designing and providing open education that is striving to shape the new environment for formal and informal education. The open learning concept embraces not only various definitions but also diverse directions conveying many opportunities for educational arrangements. Facing the need for a sustainable economy, and higher employability, governments progressively experience the pressure toward ensuring a qualified and retaining competitive workforce. There is a high demand for education settings where learners are not able to formally attend courses but experience the need to enhance their knowledge and skills. The open learning concept reflects not only educational but also business and societal issues, as well as visions and expectations. We address with this special issue innovative solutions and emerging trends in the area of open learning that comprise complex multidisciplinary fields of knowledge drawing a line between the needs of various learners in terms of accrediting current and desired level of skills and knowledge as well as of numerous institutions striving to provide education on a broad and competitive basis.

## Introduction

Advancements in Information and Communication Technology (ICT) over the last decade have lowered barriers to learner connectivity and facilitated access to a wide range of learning resources on the Web. In 2002, UNESCO adopted the term “Open Educational Resources” (OER) to refer to the “open provision of educational resources enabled by ICT”. UNESCO defined the term OER as “teaching, learning, and research resources that reside in the public domain or have been released under an intellectual property license that permits their free use or re-purposing by others. Open educational resources include full courses, course materials, modules, textbooks, streaming videos, tests, software, and any other tools, materials, or techniques used to support access to knowledge” (Atkin et al. 2007).

From the practical point of view, by being open, the content can be accessed by any learner, reused in new contexts, modified or extended for other purposes, and integrated in a shared pool of resources, and it can be the shared point of a reference for collaboration (Baytes 2014). From the social and economical point of view however, open access to OER is seen by many governments and global educational movements as the solution to the fundamental concept of the *right to education*. UNESCO’s Education for All (EFA) movement is a global commitment to provide quality basic education for all children, youth, and adults, supported, at least in principle, by 164 national governments. Nevertheless, today there are still many millions of *out-of-school* children worldwide (Pope 2014). Finally, from the technological and pedagogical point of view, though, some progress has been made in areas, such as open access research (i.e., open access publishing) and open source software provision, but many challenges in the area of policy, security, privacy, etc. are preventing this mode of learning from being properly deployed and adopted as a strategy to reduce the global education gap.

In this special issue, we address the interrelationship between technological trends, pedagogical challenges, and organizational demands emerging from this evolutionary way of learning that applied in a right way may foster education from a learner’s as well as economic perspective, helping people master various job situations and improve in the middle term the issues of employment, workforce and talent management, as well as quality of education and learning.

## Open Access framework

To justify the contributions building the basis for this special issue and previewed in the “Open access framework” section, we need firstly to clarify the general framework of an *Open Access* that serves knowledge, skills, and competency advancement in novel educational settings. This comprehensive information allows us to better identify demands and obstacles in the implementation of the open learning concept:

1. *Open access to programs* that lead to full, recognized qualifications. These are mainly offered by open universities. There are now nearly 100 publicly funded open universities around the world (Baytes 2014). These universities are often very large in terms of student numbers. Among the well-known open universities, we list The Open University in the UK which has over 200,000 students, Athabasca University and Téluq in Canada, The Open University of China (over 1 million undergraduate students and 2.4 million junior high school students), Anadolou Open University in Turkey (1.2 million students), the Open University of Indonesia (Universitas Terbuka—500,000 students), and the University of South Africa (350,000 students). These large, degree awarding national open universities provide an invaluable service to millions of students who otherwise would have no access to higher education. It should be noted however that there is no publicly funded open university in the USA, which is one reason why massive open online courses (MOOCs) have received so much attention there. Further, open, distance, and online learning are rarely found in their *purest* forms (Baytes 2014). No teaching system is completely open (minimum levels of literacy are required, for instance). Thus, there are always degrees of openness. Also, the complexity of technologies used by an open university should take into consideration the minimum entry requirements in terms of students’ computer literacy and skills. A student with low computer literacy skills may not be able to properly use advanced IT tools. So, for fairness reason and in order not to deny access to the university, students are offered to use adequate technologies. This is another reason, beside the university size, why most open universities have been behind in terms of adopting advanced technologies. Therefore, they are now increasingly challenged by both an explosion in access to conventional universities, which has taken up some of their market, and new developments such as MOOCs and open educational resources (Baytes 2014). Also, the degree completion rates of most open universities are often very low. For instance the degree completion rate for the Open University in the UK is 22 % (Woodley and Simpson 2014), but nevertheless, still higher for whole degree programs than for most single MOOC courses (Baytes 2014).
2. *Open access to courses* or *programs* that are not for formal credit, although it may be possible to acquire badges or certificates for successful completion. MOOCs are a good example. A MOOC is a model for delivering learning content online to any person who wants to take a course, with no limit on attendance, neither any restriction on learners’ academic or social background. In an MIT Technology Review, developed by Justin Pope in December 2014 (Pope 2014), it was stated that “Their interactive technology promised to deliver top-tier teaching from institutions like Harvard, Stanford, and MIT, not just to a few hundred students in a lecture hall on ivy-draped campuses, but free via the Internet to thousands or even millions around the world. At long last, there appeared to be a solution to the problem of “scaling up” higher education: if it were delivered more efficiently, the relentless cost increases might finally be rolled back.” Justin added “Some wondered whether MOOCs would merely transform the existing system or blow it up entirely. Computer scientist Sebastian Thrun, cofounder of the MOOC provider Udacity, predicted that in 50 years, 10 institutions would be responsible for delivering higher education.” However, some studies, including a high-profile experiment to use MOOCs at San Jose State University showed other conflicting results. In fact, some Faculty at San Jose and at other institutions rushing to incorporate MOOCs began pushing back, rejecting the notion that online courses could replace the nuanced work of professors in classrooms (Pope 2014). The tiny completion rates for most MOOCs drew increasing attention. On the other side, Justin also states “Other experiments have shown modest success (including a later iteration at San Jose State). In 2013, Georgia Tech announced a first-of-its-kind all-MOOC Master’s program in computer science that, at $6600, would cost just a fraction as much as its on-campus counterpart. About 1400 students have enrolled. It is not clear how well such programs can be replicated in other fields, or whether the job market will reward graduates with this particular Georgia Tech degree. But the program offers evidence that MOOCs can expand access and reduce costs in some corners of higher education.” An explanation of the small completion rate is that most learners who are enrolled in MOOCs are just curious people who are not necessarily seeking a credential. It was noticed that most of these learners drop the courses in a week or two. In Justin’s study, it was stated that most of the For-profit Coursera and edX, the nonprofit consortium led by Harvard and MIT, are up to nearly 13 million users and more than 1200 courses between them. In another study, published in September 2014, by MIT physicist David Pritchard and other researchers who developed an online course using MOOC, the authors found that the MOOC was generally effective at communicating difficult material—Newtonian mechanics—even to students who were not MIT caliber. In fact, the students who started the online course knowing the least about physics showed the same relative improvement on tests as much stronger students. “They may have started with an F and finished with an F,” Pritchard says, “but they rose with the whole class.” Pritchard still questions the effects MOOCs will have; but ideas about what they offer, and whom they might help, are evolving as rapidly as the MOOCs themselves (Pope 2014).
3. *Open educational resources* that instructors or learners can use for free. MIT’s OpenCourseware, which provides free online downloads of MIT’s video recorded lectures and support material, is one example. Many OER repositories of different nature are now made available to learners free of charge on the Web. Digital media of different sources (e.g., YouTube videos such as TED talks or the Khan Academy), and countless of OERs in the form of PowerPoint lectures, simulations, animations, and virtual worlds enable learners to access and apply knowledge and acquire skills and competencies in a wide range of disciplines. There are many thousands of examples of stand-alone, open educational resources that can be downloaded free for educational use. Examples include MIT's OpenCourseWare, Apple's iTunes University, and the UK Open University's OpenLearn. OERs are used by learners to review or master key concepts or techniques. They provide an alternative route for students who struggle to keep up in classroom lectures (Contact North 2015). They also appeal to an increasingly large group of learners who are just interested, but do not want to enroll in, a formal course or program. Another usage of OERs was adopted by the Open Educational Resources University (OERu), which is basically an international consortium of mainly British Commonwealth and US universities and colleges offering open access courses that enable learners either to acquire full credit for transfer into one of the partner universities or to build toward a full degree, offered by the university from which most credits have been acquired. Students pay a fee for assessment.
4. *Open research, textbooks, and data*: open research, whereby research papers are made available online for free download; open course textbooks that are free for students to use; and open data, that is, data open to anyone to use, reuse, and eventually redistribute. It is paramount that both students and professors should be given access to research material they need and should not be prevented from doing so because of cost. Open access to scholarly research has a significant impact on learner education. It allows them to access most relevant knowledge, techniques, and technology to develop further skills, build ability to innovate, and to make an impact in their respective fields. While most universities in developed countries provide free access to scholarly journals, many universities in developing countries do not have such facility. Therefore, the open access movement in research needs to be organized and developed further to reduce such barriers. Also, numerous publishers, both nonprofit and for-profit, voluntarily make their articles openly available at the time of publication or within 6–12 months (Swan 2010; Rossner 2010). Many have switched from a closed, subscription model to an open one as a strategic business decision to increase their journal’s exposure and impact, and have done so with great success.

This detailed framework for open access to education constitutes the key challenges as well as limitations and barriers that today’s open education faces. The following explanations serve clarifying the potential of the multidisciplinary research that is accompanied by a variety of emerging trends as specified:

1. The growing movement that is driving open access to education is characterized by the shift from a classroom-centered model based on structured curriculum and delivered through lectures to an online model based on OER repositories, accessed individually or through study groups, with an emphasis on learners supporting each other through discussion groups, new social media, and informal peer assessment.
2. The excessive use of technology addresses not only the teaching delivery but also the support and assistance for learners in terms of new forms of learner interaction and assessment, addressing aspects as social media and gaming, peer collaboration, peer assessment, work on shared projects, thus sharing experience and building on their previous knowledge.
3. A change in the role of both professor and learner is emerging. The professor is becoming the facilitator and course content developer, while the learner has developed full learner autonomy, selecting resources of their choice, and conducting online assessments at their pace. While learner autonomy has many benefits, learners are usually confused by the wide selection of OERs that are made available to them. In this situation, content expert guidance is needed.

Tackling the issues identified in the extended framework for open access to education that we outlined in this section, the next section provides an overview of the contributions in this special issue that introduce original multidisciplinary approaches, and strive to boost the value of technology to the educational environment in a systematic process by addressing the key challenge of open education, namely exploiting resources and improving organizational inadequate processes by covering learners’ needs and different learning styles, and leading to a desired business and social impact.

## Papers preview

The first paper presented in this special issue, is especially devoted to the above-listed key trend (1) of open learning, focusing on massive open online courses (MOOCs) that allow more people to gain access to education, regardless of their learning background and where content delivery is not anymore bound on standard mechanisms and physical attendance of lectures. Hughes and Dobbins undertake a remarkable research study that investigates the variables and mechanisms of how data about student performance can be captured to exploit them toward business and student performance improvement. Based on strong and robust implementation of state-of-the-art technologies, such as data warehouses, online analytical processes, machine learning and social media analytics the authors introduce methodologies and solutions that support the investigation of a student’s online behavior and course interaction to predict performance, and thus, identify at risk students before they drop-out with the aim of increasing MOOC completion rates. This is essential as capturing and analyzing data and transforming them into statistics of individuals can effectively serve both, the individual learner with his/her abilities and needs as well the organizers of the MOOC to adapt curricula, teaching processes and learning environments, simultaneously depicting a multilayered personal and business impact. The relevance of the research study presented in this paper is enormous. It provides evidence of the high demand in the MOOC application area for predictive systems within learning communities.

Chorana, Lakhdari, Cherroun, and Naoui demonstrate an original contribution to mastering the demanding situation in an open education framework where the number of students is extremely high, and e-assessment systems are in the difficult position to process a huge amount of data in terms of students’ documents and answers. This paper is simultaneously in the scope of emerging trends (1) and (2). Backed up by a critical exploration of recent technologies and approaches to automated assessment systems, the authors exploit the powerful potential of the Extensible Markup Language (XML) format and related technologies to transform both students’ and teacher’s documents to an XML format and extract from the teacher’s correct document the required skills as patterns. To assign a mark, the system measures similarities between the patterns of the students’ and the teacher’s document. Following a simple but efficient Fully Automated Assessment Approach (F3A), the resulting system can easily operate on both the student’s produced document and the teacher’s correct document without depending on any environment constraints. Starting from office skills as those most frequently applied in education and business settings, the proposed solution can be efficiently generalized to serve contemporary educational and professional organizations that need to evaluate the IT competencies and skills of a large number of persons in many exams. Therefore, it represents an effective basis to serve a variety of emerging open education forms, such as MOOCs as well as support open learning implementations, such as e-learning and learning and learner management systems.

In view of the current open education discourse, Al-Jumeily, Hussain, Alghamdi, Dobbins und Lunn present a position paper devoted to the context of all the three above-listed emerging trends in open learning. This paper focuses on an innovative research, across multiple disciplines. It discusses significant challenges and existing issues around upcoming crowdsourcing applications and intelligent adaptive systems. Giving special attention to the rapidly broadening development of open learning and open access to education, this research contributes to evolving current technology-enhanced learning approaches toward a novel open learning system by the utilization of the advantages of crowdsourcing and adaptive systems. The proposed adaptive, student-centric learning system caters for either individual or group learning and differentiates itself from other tutoring and programming support technologies due to its inherent ability to monitor and assess students’ performance in each phase of the education process. Thus, it is especially appropriate for teaching and enhancing programming skills, building software, and then as necessary executing, debugging, and correcting the solutions. Furthermore, it addresses the demand for appropriately designed intelligent systems that serve various learners, in terms of distinctive available and/or desired skills and knowledge. The proposed framework underpins the merge between an intelligent adaptive system and crowdsourcing approaches to be beneficially applied toward the improvement of teaching processes and programming skills, especially very useful for novice programmers, in an open learning context, combining the outcomes of traditional, and promising ground-breaking research to serve today’s needs. Against this background, the authors pioneer a new line of research and development in novel open education settings.

## Conclusion

The open learning concept appears as a novel approach of people following a dream that a *better technology*-driven process to education will overcome local institutional legacy systems, non-compatible security and privacy policies, and sometimes non-available technological interoperability. Researchers, academics, experts, and policymakers will further explore, and shape the scope of the open learning concept based on latest regional, economic and technology developments. We believe to facilitate with our findings, the important experiences, approaches, and recommendations provided in this special issue of the RPTEL Journal, the adaptive implementation of the open education concept to advance the educational landscape and the employability of people and maximize the institutional benefit. We aimed to create awareness and better understanding of the complex interrelationship between institutional and learner’s needs in an challenging open education eco-system that allows more people to gain access to education, regardless of their learning background.

The contributions in this special issue provide strong evidence that further innovative research is needed to identify the key success factors that are to be followed and measured in order to ensure an overall impact of this promising education form. These success factors have to consider the learner’s needs and abilities, a rarely employed approach by now, to enhance pedagogical solutions toward reflective learning and assessment as well as deeply embody privacy and security matters into organizational work to effectively exploit personal data for the benefit of all, users and the organization.

In following this line, we would like to thank the authors for their contributions toward exploiting recent limitations of open learning solutions and offering far-reaching pioneering approaches to overcome the issues and help shaping future education for all. We would also like to thank our reviewers for their comments and suggestions. We highly admire the opportunity the Editorial Board of the RPTEL Journal provided to support this special issue and particularly, its scope on highlighting the remarkable resources of the open learning concept for advanced multidisciplinary research set up on the cross-border between pedagogy, technology, and economic impact.
